# Editorial: The role of nano-therapeutics in precision cancer medicine

**DOI:** 10.3389/fbioe.2026.1818253

**Published:** 2026-03-19

**Authors:** Shue Wang, Trung Thach, Alessandro De Vita

**Affiliations:** 1 Department of Chemistry, Chemical and Biomedical Engineering, University of New Haven, West Haven, CT, United States; 2 Department of Biological Sciences, Purdue University, West Lafayette, IN, United States; 3 Preclinic and Osteoncology Unit, Biosciences Laboratory, IRCCS Istituto Romagnolo per lo Studio dei Tumori (IRST) “Dino Amadori”, Meldola, Italy

**Keywords:** cancer immunotherapy, CRISPR/Cas delivery, drug delivery, nanomedicine, nano-therapeutics, precision oncology, tumor microenvironment

Precision cancer medicine increasingly requires therapeutic systems that can do more than simply carry a payload. Effective interventions must cross biological barriers, adapt to tumor heterogeneity, exploit tumor microenvironment vulnerabilities, and often coordinate cytotoxic and immunomodulatory effects within a controllable therapeutic window. In this context, nano-therapeutics are evolving from passive carriers into programmable therapeutic systems that integrate targeting, release control, and multimodal biological activity. The present Research Topic provides a timely overview of this transition and highlights how nanotechnology is being repositioned as an enabling discipline for precision oncology (Zou et al.; Mulè et al.; Liu et al.; Wang et al.; Zou et al.).

This Research Topic includes five articles, with four published papers and one review accepted in February 2026 (Zou et al.; Mulè et al.; Liu et al.; Wang et al.; Zou et al.). Although diverse in scope, these contributions converge on a shared objective: improving therapeutic precision through rational delivery design. The papers address key translational challenges, including the formulation of poorly bioavailable anti-cancer compounds, the orchestration of immune activation and checkpoint modulation, and the controlled transport of advanced biologic payloads such as CRISPR/Cas components and immune agonist prodrugs. Taken together, they show that the field is moving beyond simple formulation optimization toward integrated therapeutic engineering.

Mulè et al. present an original study on PLGA nanoparticles for capsaicin delivery in HepG2 cells, addressing a classic limitation of natural-compound therapeutics: poor bioavailability and limited pharmaceutical usability (Mulè et al.). Capsaicin has recognized anti-tumor potential, but its development is constrained by short half-life and dose-limiting adverse effects. By optimizing PLGA nanoparticle preparation, the authors report very high encapsulation efficiency and demonstrate improved pro-apoptotic activity *in vitro*, including increased caspase 3 activity and reactive oxygen species generation in HepG2 cells compared with free capsaicin. The study is notable not only for formulation performance, but also for linking nanoparticle engineering to biologically meaningful cellular readouts in a tumor-relevant model.

Liu et al. contribute a multifunctional immuno-nanotherapeutic strategy in a murine hepatocellular carcinoma model using nanobubbles co-loaded with shikonin, an inducer of immunogenic cell death, and miR-497-5p, used to suppress PD-L1 expression (Liu et al.). Following ultrasound-triggered activation, the platform combines induction of danger signals associated with immunogenic cell death with checkpoint-pathway modulation. The authors report strong tumor inhibition and describe immune activation features consistent with enhanced antigen presentation and macrophage activation. This study exemplifies the growing importance of externally triggerable nanoplatforms that integrate drug delivery, immune priming, and checkpoint regulation in a single therapeutic design.

At the level of enabling technologies, Wang et al. review advances in nanomaterial-mediated CRISPR/Cas delivery, from lipid nanoparticles to vesicle-derived systems (Wang et al.). The review provides a comparative framework across lipid-based, polymeric, inorganic, and extracellular-vesicle-inspired carriers, emphasizing how material properties shape targeting, endosomal escape, intracellular trafficking, and editing performance. Of particular relevance to precision cancer medicine is the discussion of how surface ligands, charge tuning, PEGylation, and stimuli-responsive behavior influence biodistribution and on-target activity while reducing immune responses and off-target effects. The review also highlights ethical and regulatory considerations and stresses the need for stronger *in vivo* potency studies, improved biocompatibility assessment, and standardized manufacturing, all of which are decisive for clinical deployment of *in vivo* editing approaches.

Zou et al. further expand the immunotherapy landscape with a review on covalent organic frameworks (COFs) for cancer immunotherapy (Zou et al.). The article emphasizes the value of COFs as structurally tunable nanoplatforms capable of supporting tumor microenvironment remodeling, multimodal therapy sensitization, and controlled delivery of immunotherapeutic payloads. By discussing hypoxia alleviation, glutathione depletion, photodynamic and sonodynamic sensitization, and immunogenic cell death induction, the review illustrates how COF engineering can be used to amplify anti-tumor immune responses. The authors also discuss advanced applications such as imaging-guided therapy, tertiary lymphoid structure induction, and abscopal-effect activation, underscoring the ambition of next-generation nanoplatforms to shape systemic immune outcomes rather than local drug deposition alone.

A complementary and highly timely contribution is the accepted review by Zou et al. on precise delivery and controlled release strategies for TLR7/8 agonist prodrugs in cancer immunotherapy (Zou et al.). TLR7/8 agonists such as resiquimod are potent immune adjuvants, but their development is often limited by systemic inflammatory toxicity. The review outlines how prodrug chemistry and smart delivery systems can jointly enable tumor-selective activation in response to microenvironmental triggers, improve spatiotemporal control, and support co-delivery with antigens, siRNA, or chemotherapeutic agents. Particularly important is the conceptual integration of chemistry and nanotechnology: the manuscript frames prodrug design not as a stand-alone masking strategy, but as part of an overall therapeutic architecture in which release kinetics, biodistribution, and immune context determine efficacy and safety.

Taken together, the five contributions point to three major directions for the field. First, precision in nanomedicine now depends on controllable delivery and biological programming, not passive accumulation alone. Second, platforms are becoming more integrated, combining cytotoxic, immunologic, and gene-regulatory functions within a single therapeutic architecture. Third, translational relevance requires parallel development of efficacy, safety, reproducibility, scalability, and manufacturability. This shift is essential if nano-therapeutics are to move from elegant proof-of-concept systems to clinically robust precision interventions.

This translational emphasis also resonates with prior work from De Vita and colleagues, including contributions linked to IRCCS IRST “Dino Amadori”, which have helped bridge nanotherapeutic innovation and clinically meaningful oncology models. In triple-negative breast cancer, De Vita et al. reported lysyl oxidase-targeting engineered lipid nanovesicles with enhanced anti-tumor efficacy, prolonged survival, and improved biocompatibility in preclinical settings, illustrating the promise of microenvironment-oriented nanotherapeutic design (De Vita et al., 2021a). In soft tissue sarcoma, De Vita et al. also showed the value of patient-derived tridimensional models and zebrafish systems to study trabectedin activity and extracellular matrix involvement, reinforcing the importance of biologically relevant platforms for treatment stratification and translational interpretation (De Vita et al., 2021b). In parallel, the IRST-associated review by Mercatali et al., co-authored by De Vita, provides a broader framework for positioning nanotechnology in sarcoma management and highlights opportunities for applying smart nanoplatforms to rare and heterogeneous tumors (Mercatali et al., 2022).

In conclusion, this Research Topic highlights the maturation of nano-therapeutics into adaptable platforms for precision cancer medicine. Beyond improved pharmacokinetics or payload protection, the studies collected here illustrate a broader transition toward programmable interventions that synchronize chemistry, carrier design, immune modulation, and disease-context specificity. Continued progress will depend on close collaboration among materials scientists, cancer biologists, immunologists, and translational clinicians, as well as on rigorous preclinical models and manufacturing-aware development strategies. The contributions assembled in this Research Topic provide a strong and timely foundation for that next phase.
